# Angiogenesis Changes in Ovariectomized Rats with Osteoporosis Treated with Estrogen Replacement Therapy

**DOI:** 10.1155/2019/1283717

**Published:** 2019-07-04

**Authors:** Yige Zhang, Fei Hua, Kai Ding, Haifeng Chen, Chenyang Xu, Wenge Ding

**Affiliations:** Department of Orthopaedics, Third Affiliated Hospital of Soochow University, Changzhou 213003, China

## Abstract

To investigate whether angiogenesis changes in early menopausal osteoporosis treated with estrogen replacement therapy, 120 rats were randomly divided into five groups: sham operation group (SHAM), ovariectomy group (OVX), and ovariectomy plus three different estrogen doses replacement therapy groups (OVX + E2). We detected the bone microarchitecture and measured the expression levels of estrogen receptor beta (ER*β*), vascular endothelial growth factor (VEGF), osteoprotegerin (OPG), and receptor activator of NF-*κ*B ligand (RANKL). CD31 immunofluorescence and silica gel perfusion imaging were used to analyze the vascular distribution. We confirmed that the femur BMD of ovariectomized rats was significantly lower than SHAM group and OVX+E2 groups. After estrogen therapy, the local microvascular formation increased after estrogen treatment in a dose dependent manner. ER*β* was downregulated and VEGF was upregulated, positively correlated with estrogen dosage. We successfully constructed an osteoporosis model of ovariectomized rats with estrogen replacement therapy. We also found angiogenesis changed in early menopausal osteoporosis treated with estrogen replacement therapy. We indicated that estrogen replacement therapy increased angiogenesis through VEGF upregulation. However, we observed that, at the highest doses of estrogen studied, increased angiogenesis was associated with a decrease in BMD, the underlying mechanisms of which remain unclear.

## 1. Introduction

Osteoporosis is a very common disease. About 30% of 50-60 years-old women in China have postmenopausal osteoporosis, and the prevalence rate in women over the age of 60 is about 30% -50%. The decline rate of bone mineral density (BMD) in postmenopausal women is three times higher than premenopausal women [1]. For older women, hip fracture and lumbar vertebrae fracture are the most serious consequences from osteoporosis and are also among the most likely causes of disability [2]. The World Health Organization (WHO) set October 20th as the “World Osteoporosis Day”, in order to draw people's attentions to osteoporosis. Therefore, the prevention and treatment of postmenopausal osteoporosis in women are very important.

In recent years, more and more studies have focused on the relationship between angiogenesis and osteoporosis, since osteogenesis and angiogenesis are closely related [3]. The currently accepted relationship between osteoporosis and angiogenesis is that the number of local blood vessels was significantly reduced after ovariectomy [4] and the decrease of angiogenesis leads to osteoporosis and the increase of local angiogenesis can relieve osteoporosis [5]. The artificially increased angiogenesis indeed alleviates the degree of osteoporosis, but it does not provide a more in-depth understanding of the mechanisms of postmenopausal osteoporosis and estrogen replacement therapy.

In addition, estrogen is known to play a significant role in angiogenesis, and it was once considered as a protective factor for blood vessels [6]. In the treatment of estrogen receptor-positive breast cancer, selective estrogen receptor modulators can effectively inhibit angiogenesis [7]. During the ischemia-reperfusion injury of lower extremity muscles, estrogen receptor *β* (ER*β*)-mediated pathways can promote blood vessel formation [8]. Previous studies have confirmed that estrogen receptors are closely related to angiogenesis in a variety of cancers and cardiovascular diseases, such as breast cancer [9, 10]. However, two randomized prospective trials showed that although estrogen replacement therapy could reduce osteoporosis in postmenopausal women, it increased the risk of cardiovascular diseases; especially in the premenopausal period, whether women need to take estrogen supplement is still controversial.

We believe that, in the widely used estrogen replacement therapy for postmenopausal osteoporosis, estrogen acts on osteoporosis by affecting angiogenesis. However, how estrogen affects osteoporosis development and angiogenesis is still unclear. In this study, we compared the vascular changes and bone microstructure of ovariectomized rats treated with different doses of estrogen and analyzed the protein expression of the genes related to angiogenesis and osteoclast in local bone tissue, in order to explore how estrogen affects osteoporosis and angiogenesis in ovariectomized rats, providing new ideas for estrogen replacement therapy and the prevention of related complications.

## 2. Materials and Methods

### 2.1. Animal

The animal protocol was approved by the Animal Research Committee of Suzhou University. All the animals were handled in accordance with the guidelines of Institutional Animal Care and Use Committee at Suzhou University.

120 SD female rats at 6 months old, weighing 280-300 g, were randomly divided into sham operation group (SHAM), ovariectomy group (OVX), and ovariectomy plus estrogen replacement therapy group (OVX+E2); the OVX+E2 group was further divided into three groups based on estrogen dose: high dose (50 ug/kg/time) group (H), normal dose (20 ug/kg/time) group (N), and low dose (5 ug/kg/time) group (L)[11]. In total, 5 groups were studied with 24 rats in each group. Estrogen injection is prepared by dissolving *β*-Estradiol (Solarbio, E8140) in sterile olive oil. Estrogen injection was started three days after ovariectomy, performed every other day. SHAM and OVX groups received same volume of vehicle. All rats were anesthetized using 30 mg/ml sodium pentobarbital (10 mg) via intraperitoneal injection. Routine preparation was conducted before surgery. For ovariectomy, a longitudinal incision about 1.0 cm long was made in the middle of the rat back, and the back skin was cut open. After the skin, fascia and muscles were separated to the sides; the abdominal cavity was open from the thinner layer of posterior abdominal wall on both sides. Then, the ovary was ligated with silk and removed, and the incision was closed. After surgery, the animals were placed in the cage, with free access to food and water. The rats were given penicillin G (40000 U/kg/day) by intraperitoneal injection for 3 days. 1 week later, the suture was removed. All the animals were raised at the SPF Animal Breeding Center of Suzhou University, with free access to sterile pellet feed (Ca: 0.95%; P: 0.67%) and sterile water. Room temperature was controlled at 22°C or so, and the relative humidity was controlled at about 56%. 12 hours of light and night were alternated, with regular UV disinfection and ventilation.

### 2.2. Experimental Design

All the rats in each group were anesthetized by intraperitoneal injection of 30 mg/ml sodium pentobarbital (10 mg) at 6 weeks after surgery and then sacrificed. The rats were restricted from food and water at the night before execution. Among the 24 rats in each group, 18 were sacrificed by cervical dislocation, and their femurs were collected after removing the surrounding soft tissues. The femurs from 12 rats were wrapped with gauze soaked with PBS and stored at -20°C, 6 of which are for the use of bone mineral density measurement and micro-CT analysis and the rest are for frozen section; the femurs from remaining 6 rats were used to extract protein for further analysis. The other 6 rats were sacrificed by abdominal aorta bleeding. After silicone rubber (Microfil) perfusion, the femur fracture specimens were fixed and decalcified, followed by micro-CT scan to examine microvascular structures.

### 2.3. Femur Bone Mineral Density, Mciro-CT Scan, Three-Dimensional Reconstruction, and Morphometry Analysis

Micro-CT (SCANCO Medical AG, *μ*CT100) was used to detect the microstructure of femur specimens. The isolated femur specimens were fixed on micro-CT scanning platform. The specific parameters are as follows: tube voltage 70 kV, tube current 200 uA, layer thickness 20 um, exposure time 300 ms, and 360-degree rotation scan. The proximal part of femur was selected for scanning, and the standard phantom was scanned at the same time in order for CT correction. After the scan, we selected the center cylindrical bone tissue of femur neck as our region of interest (ROI), with the diameter of 2mm and the thickness of 1 mm. The bone tissues within ROI were analyzed by 3D visualization. Quantitative analysis of bone mineral density (BMD) was conducted by the built-in scanco microCT *μ*100 evaluation. The 3D structural analysis parameters included bone volume fraction, BV/TV (%), average trabecular thickness (Tb. Th), mean trabecular separation (Tb. Sp.), and trabecular number (Tb.N.). Each specimen was tested three times, and the variation coefficients were less than 2% within or between groups.

### 2.4. Bone Frozen Sections and Hematoxylin & Eosin (HE) Staining

The proximal end of femur was fixed with 4% paraformaldehyde at 4°C for 48 hours, followed by rapid decalcification by decalcifying solution for 24 hours. The decalcification endpoint was judged by whether the bone tissue is soft enough to pin in. Then, the decalcification solution was removed, and the tissue was washed with saline. The specimens were then sectioned on a cryostat. The histology staining followed the regular steps: deparaffinization with xylene, hydration with gradient alcohol solutions and distilled water, hematoxylin and eosin staining, dehydration, cleared, and mounting. The proximal femur sections were observed under optical microscope.

### 2.5. Vascular Perfusion

Microfil contrast agent (Flow Tech, Carver, MA, USA) was mixed at the required ratio according to instructions. Contrast agent consisted of 42% Microfil MV-122, 53% diluent and 5% fixative. After the rat was anesthetized with 30 mg/ml sodium pentobarbital (10 mg), its abdominal cavity was opened, and the abdominal aorta catheterization was conducted from the top of femoral artery branch. A small incision was made on the inferior vena cava to form a circulatory pathway. About 250 ml saline was perfused via aortic catheter until the effluent was transparent. Then, the perfusion buffer was changed to 250 ml of 10% formalin, followed by contrast agent, allowing the contrast agent to distribute in the main femoral vascular and its branches. The perfusion was successful when the toes of rat hindlimb were filled with yellow contrast agent. After the contrast agent perfusion, the animals were stored in a refrigerator at 4°C overnight. Then, femur specimens were further fixed in 10% formalin for about 1 week and thoroughly decalcified before micro-CT scan.

### 2.6. Western Blot

Freshly isolated proximal rat femur was placed in the mortar and ground with liquid nitrogen at low temperature. The bone tissue was lysed on ice to extract total protein. BCA method was used to determine protein concentration. 20 *μ*g protein from each sample was loaded on SDS page, followed by electrophoresis, transfer, and blocking at room temperature for 1 h. Primary antibody was used at 1: 500, and secondary antibody was rabbit anti-mouse IgG. *β*-actin was used as the internal control. The film was developed and analyzed by Image J. The relative expression level of target protein was defined as the ratio of target band intensity to the internal control band intensity.

### 2.7. Immunofluorescence

The frozen sections were fixed with acetone (ice cold acetone) at room temperature for 10 min and washed with PBS for 3 × 5 min. Then the slides were blocked with 2% BSA at 37°C for 60 min and placed in a wet box. CD31 antibody (1: 100, abcom) was added and incubated overnight at 4°C. Next day, the slides were recovered at room temperature for 1 hour and then rinsed with PBS for 3 × 20 min. Secondary antibody goat anti-mouse IgG (1: 500; Sigma) was added and incubated at 37°C for 60 min, followed by PBS wash for 3 × 10 min. The nucleus was stained with dapi for 10 min, followed by PBS wash for 3 × 5 min. Finally, the slides were mounted using antifluorescence quenching solution and observed under fluorescence microscope. All the steps were operated in dark once the fluorescent secondary antibody was added.

### 2.8. Statistical Analysis

All of the experiments were repeated independently at least three times. All data are presented as mean ± standard deviation (SD). SPSS 23.0 and one-way ANOVA were used to compare between groups. LSD (Least-Significant Difference) was performed as post hoc testing to compare the between-group differences presented in the results. P< 0.05 is considered statistically significant.

## 3. Results

### 3.1. Femoral Bone Mineral Density and Morphological Parameters of Each Group

**Figure 1:**
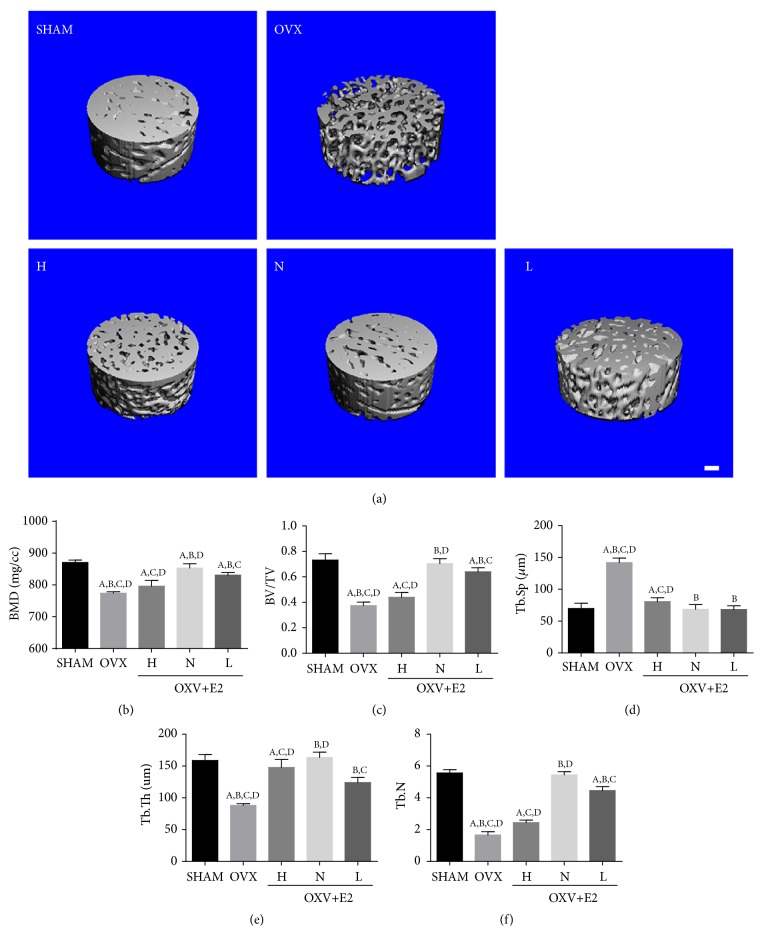
*MicroCT of femurs*. (a) MicroCT 3D reconstruction of region of interest of femoral head (n=6/group; scale bar, 100 *μ*m). (b) BMD in the five groups. (c) The bone volume fraction (BV/TV) in the five groups. (d) The trabecular separation (Tb.Sp) in the five groups. (e) The trabecular thickness (Tb.Th) in the five groups. (f) The trabecular number (Tb.N) in the five groups. Values are means ± SD of 6 mice in each group. A, P<0.05 versus sham group; B, P<0.05 versus OVX + E2 (H) group; C, P<0.05 versus OVX + E2 (N) group; D, P<0.05 versus OVX + E2 (L) group.

The 3D images of region of interest of femoral neck are presented in Figure 1(a). The bone mineral density (BMD) of OVX group was significantly lower than SHAM group. After exogenous estrogen supplementation, the BMD reduction was effectively alleviated in N group and also moderately enhanced in L group. However, the BMD of H group was only slightly improved, showing significantly less effect than N group and L group (Figure 1(b)). Femur micro-CT showed that the bone mass of OVX group was significantly decreased, and the decline was markedly improved after different doses of estrogen treatments. Analysis of bone parameters showed that BVF (Figure 1(c)), TB.Th (Figure 1(d)), Tb.Sp (Figure 1(e)), and Tb.N (Figure 1(f)) of OVX group all exhibited different degrees of osteoporosis features; estrogen treatment improved the bone parameters, including the increased BVF, Tb.Th, and TB.N and reduced Tb.Sep. The bone loss in the N and L groups was significantly improved compared with the OVX group, while the H group was slightly improved compared with the OVX group, and the H group was significantly different from the N and L groups.

### 3.2. Hematoxylin & Eosin (HE) Staining

**Figure 2:**
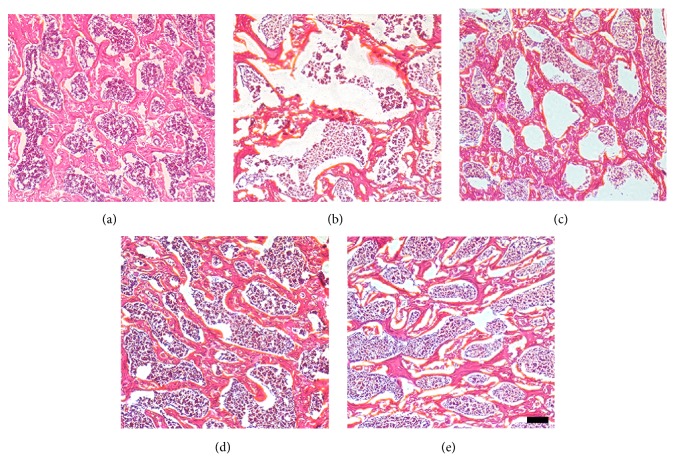
*H&E staining images*. (a) H&E staining of sham group; (b) H&E staining of OVX group; (c) H&E staining of OVX + E2 (H) group; (d) H&E staining of OVX + E2 (N) group; (e) H&E staining of OVX + E2 (L) group (n = 6/group; scale bar, 100 *μ*m).

The HE staining of rat femur is shown in Figure 2. The trabecular bone of SHAM and OVX+E2 groups was relatively more abundant, with better structural arrangement and continuity. The trabeculae of OVX group showed decreased continuity compared to SHAM group. And the continuity of trabecular in OVX+E2 group was better than OVX group. Although there was no significant difference in continuity among H, N, and L groups, the trabecular separation of the H group was greater than that of the N group and the L group, and the fat vesicles in the bone marrow were more than the N group and the L group. The trabecular thickness H and N L group was significantly greater than that of L group.

### 3.3. Changes in Femoral Protein Levels

**Figure 3:**
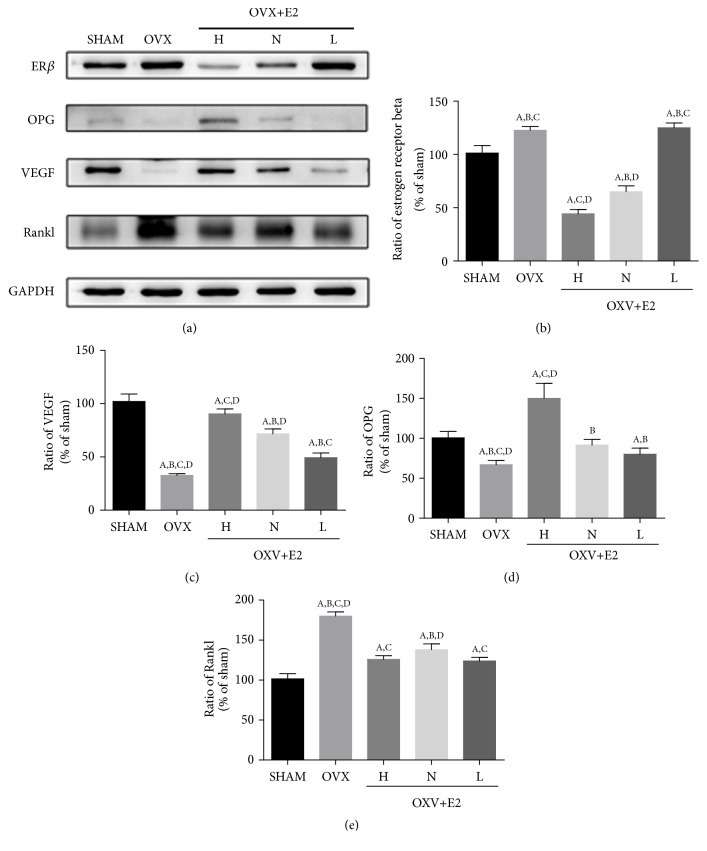
*Changes in femoral protein levels*. ER*β*, VEGF, OPG, and Rankl protein expression changes in femurs (a). ER*β* (b), VEGF (c), OPG (d), and Rankl (e) protein expression was quantified with densitometry. Values are means ± SD of 6 mice in each group. A, P<0.05 versus sham group; B, P<0.05 versus OVX + E2 (H) group; C, P<0.05 versus OVX + E2 (N) group; D, P<0.05 versus OVX + E2 (L) group.

Western blot results are shown in Figure 3(a). Western blot showed that the RANKL protein level was significantly elevated in OVX group compared to SHAM group, and was markedly reduced after estrogen treatments, although there was no significant difference between different doses (Figure 3(e)). The OPG protein levels in H and N groups were significantly higher than OVX group (Figure 3(d)), and L group showed no difference. After exogenous estrogen supplementation, the expression of ER*β* was significantly reduced in H and N groups compared to OVX groups (Figure 3(b)), and L group showed no difference. The expression of VEGF was significantly decreased in OVX group compared to SHAM group but was significantly increased after estrogen treatment (Figure 3(c)).

### 3.4. Microfil Vascular Perfusion Imaging

**Figure 4:**
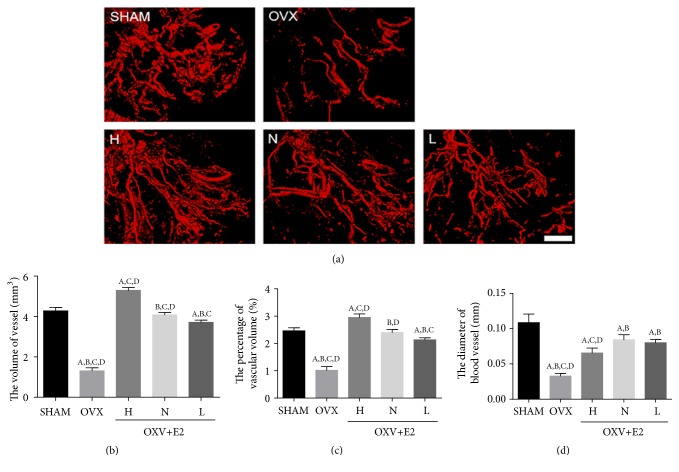
*MicroFil Angiography of femurs*. (a) Micro-CT reconstruction and parameters of femoral artery of five groups (n = 6/group; scale bar, 1.0 mm). (b) The volume of vessel in the five groups. (c) The percentage of vascular volume in the five groups. (d) The diameter of blood vessel in the five groups. Values are means ± SD of 6 mice in each group. A, P<0.05 versus sham group; B, P<0.05 versus OVX + E2 (H) group; C, P<0.05 versus OVX + E2 (N) group; D, P<0.05 versus OVX + E2 (L) group.

The perfusion imaging (Figure 4) showed that the mean vascular volume, vascular diameter, and vascular fraction of OVX+E2 group were all significantly higher than OVX group in a dose dependent manner: H group showed higher vascular parameters than N group or L group.

### 3.5. CD31 Immunofluorescence

**Figure 5:**
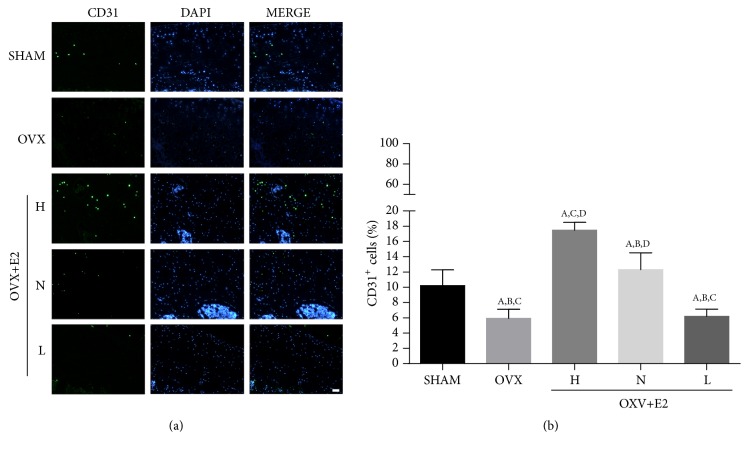
*Immunofluorescence of CD31*. (a) Immunofluorescence of CD31 and DAPI in the femur neck of five groups (n = 6/group; scale bar, 100 *μ*m). (b) The percentage of cells expressing CD31 in five groups.

The CD31 immunofluorescence on frozen femur sections is shown in Figure 5(a). The amount of CD31 staining was significantly lower in OVX group compared to SHAM group. H group exhibited higher CD31 staining than other groups, indicating that the local blood vessel formation was increased in H group. The CD31 staining was also increased in N group and L group, but L group was not significantly higher than OVX group (Figure 5).

## 4. Discussion

Osteoporosis is a disease characterized by decreased bone mass and degeneration of bone tissue. The decline of estrogen level plays a critical role in the disease initiation and progression. The traditional view is that estrogen can directly inhibit the activation of osteoclasts through OPG/RANKL pathway, thus alleviating osteoporosis. Our study found that angiogenesis changed in early menopausal osteoporosis treated with estrogen replacement therapy and the effect of estrogen replacement therapy in ovariectomized rats might be mediated by increased local angiogenesis. This function might be independent of inhibiting osteoclast activity and was possibly related to ER*β*-mediated changes in VEGF. In addition, the promotion of angiogenesis by estrogen is not completely beneficial. When estrogen is overdose, angiogenesis is increased, but bone density is lower than that the normal dose group. The specific mechanism needs further investigation.

In our study, we successfully constructed an osteoporosis model of ovariectomized rats with estrogen replacement therapy. Our results confirmed the fact that estrogen can inhibit osteoclasts through OPG/RANKL pathway in the early menopause, thereby improving the bone loss. However, no bone mass loss was observed in patients receiving estrogen replacement therapy during menopause [12]. The bone tissue mirco-CT showed that the estrogen replacement at low dose and normal dose could significantly improve the local bone loss after menopause. Previous studies have analyzed the histopathological structures of bone tissues from ovariectomized rats under both low dose and normal dose estrogen treatments, but did not find significant difference in improving bone loss between these two treatments [13]. An analysis of the WHI performed assessing risk by age indicated that women who took hormone therapy closer to the age of menopause had a decreased risk of coronary heart disease, compared to those who took hormone therapy at older ages, although the trend did not meet statistical significance [14]. Moreover, the increasing estrogen concentration also brings higher risks of breast cancer and venous thrombosis; exclusive use of estrogen can increase the risk of endometrial cancer, which is positively correlated with serum estrogen levels. These risks brought by estrogen replacement therapy seem to closely relate to blood vessels. Therefore, by elucidating the effects of estrogen on angiogenesis in local bones, we can have a better understanding in estrogen replacement therapy and the prevention of complications from the mechanistic perspective.

The primary trigger for skeletal vascularization is the formation of vascular networks induced by hypoxia-inducible factor (HIF-1) mediated VEGF expression in skeletal microenvironment [3]. Weng et al. showed that during bone repair, conditional activation of VEGF by HIF-1 pathway could increase angiogenesis and prevent age-related bone loss [15]. The artificially increased angiogenesis indeed alleviates the degree of osteoporosis, but it does not provide a more in-depth understanding of the mechanisms of postmenopausal osteoporosis and estrogen replacement therapy. So far, no study has investigated the effect of early estrogen replacement therapy on the relationship between angiogenesis and osteoporosis. Our study, for the first time, examined angiogenesis change in early menopausal osteoporosis treated with estrogen replacement therapy. By performing CD31 immunofluorescent on bone frozen sections and micro-CT to observe microvascular structures, we found that exogenous estrogen supplementation could increase local blood vessel formation in a dose dependent manner. The results showed that early estrogen supplementation could indeed changing the local angiogenesis in rat with osteoporosis by ovariectomized, which, to some extent, explained why the effect of estrogen on osteoporosis became stronger with increasing doses. Moreover, our results showed that the enhanced angiogenesis mediated by estrogen might not depend on the inhibitory effect of estrogen on bone destruction, but due to other independent protective mechanisms. In many other tissues, estrogen also plays an important role in angiogenesis. In adipose tissue, the angiogenesis mediated by estrogen receptor and VEGF is involved in the regulation of various physiological functions of adipose tissue [16]. Low dose estrogen usually induces angiogenesis through HIF pathway in hypoxic environment. Previous study has shown that high dose estrogen can also promote angiogenesis under normoxia state [17]. However, it is surprising that although the bone destruction was significantly improved with different doses of estrogen supplementation, the bone mineral density of high dose group was lower than normal dose group. It is possible that the high dose estrogen may lead to elevated plasma creatinine levels, severe proteinuria, tubule dilatation, tubulointerstitial injury, hydronephrosis, glomerulosclerosis, and oxidative stress, which affects calcium and phosphorus metabolism [18]. Estrogen usually plays a protective role for blood vessels. However, after the formation of arterial plaque, the presence of estrogen may promote plaque rupture and shedding by activating inflammatory reactions, which is detrimental to local microcirculation [19]. Therefore, although the angiogenesis was significantly increased in high dose estrogen group, the possibility of vascular damage was also increased.

We speculate that the effect of estrogen on angiogenesis during postmenopausal osteoporosis might result from altered VEGF mediated by ER*β*. Some studies showed that the protective effect of estrogen on blood vessels was mainly mediated by ER*β* [6], suggesting that ER*β* plays an important role in angiogenesis of postmenopausal osteoporosis. Another study suggested that the presence of ER*β* is associated with vascular damage [20]. In humans, ER*β* expression increases with aging, which is positively correlated with the production of several proinflammatory markers [21]. In our study, we found that ER*β* decreased in local bone tissue, together with increased VEGF level and angiogenesis. These results suggest that the increased angiogenesis induced by estrogen may be mediated by the inhibition of vascular injury. In retinal cells, Giddabasappa et al. found that, by activating ER*β*, the formation of pathological blood vessels in the retina is inhibited under hypoxia conditions [22]. HIF-1a plays an important role in both bone formation and bone angiogenesis. Liu et al. found that, in HIF-1a conditional knockout mouse, bone formation and angiogenesis were both significantly reduced [23]. Combined with previous experimental results, it is likely that ER*β* has a major impact on angiogenesis in osteoporosis under the hypoxia condition induced by HIF-1a. In addition, some studies have successively knocked out ER*β* in osteoblasts, osteoclasts, and osteocytes or two populations together. Surprisingly, they found no significant changes in the bone mass of female rats [24]. Therefore, ER*β* not only affects bone formation by acting on osteogenesis or osteolysis in the bone microstructure, but also may change bone formation through other means.

In this study, we observed the alterations in bone microvascular structures and vascular densities in rats after exogenous estrogen supplementation and examined the changes of ER*β* and VEGF proteins. We have to point out that we did not fully investigate whether ER*β* is directly related to angiogenesis after exogenous estrogen supplementation, but we proposed a possible pathway through analyzing protein expression and vascular morphology. In our opinion, estrogen may affect osteoporosis through ER*β*-related angiogenesis. With the increase in estrogen dosage, the formation of blood vessels in the local bone is increased. However, when estrogen is overdose, the bone mineral density is lower than normal dose treatment, of which the specific underlying mechanism needs further investigation. Next, we will focus on studying the impact of ER*β* on the local vascularization during postmenopausal osteoporosis.

## Data Availability

The data used to support the findings of this study are available from the corresponding author upon request.
